# A matter of quantity: The effect of chloroplast stromal phosphate levels on photosynthetic efficiency

**DOI:** 10.1093/plphys/kiae307

**Published:** 2024-05-28

**Authors:** Pablo Ignacio Calzadilla

**Affiliations:** Assistant Features Editor, Plant Physiology, American Society of Plant Biologists; Instituto de Fisiología Vegetal (INFIVE), Universidad Nacional de La Plata—CONICET, cc 327, 1900 La Plata, Buenos Aires, Argentina; Department of Earth and Environmental Sciences, Faculty of Science and Engineering, University of Manchester, Manchester, M13 9PT, UK

Phosphorus (P) is an essential mineral nutrient for plants, playing central roles in development, growth, and environmental responses. In the soil, P is available in the form of inorganic phosphate (Pi), which, once incorporated by plants, can be converted into nucleic acids, membrane lipids, and sugars. Pi is also necessary for ATP synthesis during photosynthesis, and its concentration in the chloroplast is linked to photosynthetic regulation. Environmental fluctuations and metabolic changes can alter chloroplastic Pi homeostasis, triggering acclimation responses to maintain photosynthetic performance (Bouain et al. 2022). Stromal Pi levels need to be high enough to sustain photophosphorylation without inhibiting Calvin-Benson cycle reactions (Grotjohann and Gräber 2002; Marcus et al. 2005). Therefore, plants strictly regulate Pi levels to avoid inhibition of photosynthesis.

The concentration of Pi in the chloroplast stroma is determined by photophosphorylation rates, Pi recycling from the Calvin-Benson cycle and starch synthesis, and Pi import from the cytosol. The latter involves a series of molecular transporter families: plastidic phosphate translocator (pPT), PHOSPHATE TRANSPORTER 2 (PHT2), and PHT4 (Versaw and Garcia 2017). In particular, the triose phosphate/Pi translocator (TPT, a member of the pPT family) couples Pi import to the stroma with triose phosphate export (produced by photosynthesis) to the cytosol (Fliege et al. 1978; Flügge et al. 1989) (Fig.). Since TPT is fundamental for carbon export to the cytosol, it is expected to be one of the main pathways for Pi translocation to the chloroplast stroma. However, *tpt* mutants are not growth impaired, suggesting that additional Pi transport mechanisms and alterations in starch metabolism might be involved in Pi chloroplast homeostasis and triose phosphate export (Walters et al. 2004). In this regard, PHT2 and PHT4 transporters could complement the deficiency of TPT-mediated Pi transport in the *tpt* mutants. However, the contribution of these transporters to stromal Pi levels and their regulation is poorly understood.

**Figure.**
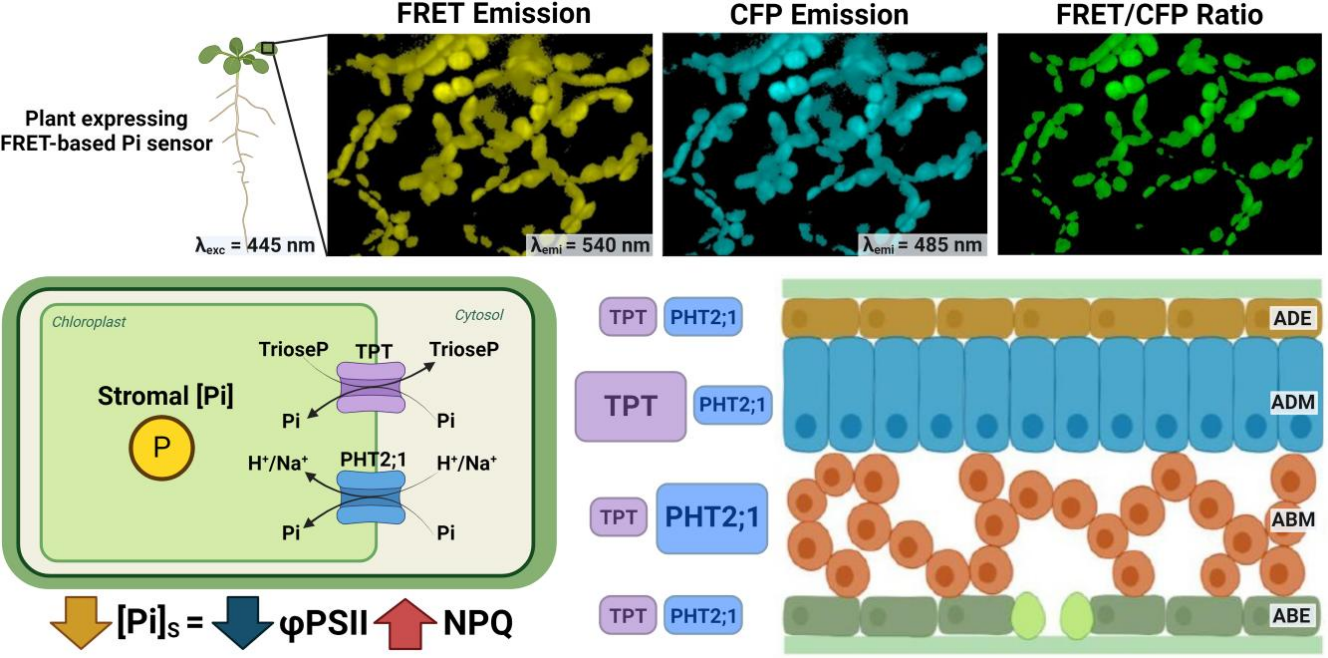
Low chloroplast stromal Pi levels impair photosynthetic performance in *Arabidopsis thaliana*. A FRET-based sensor was used to estimate stromal Pi levels in the chloroplasts. Low Pi levels increase FRET emission over the donor CFP fluorescence emission (increasing FRET/CFP ratio). By contrast, high Pi decreases the FRET/CFP ratio. Using the FRET sensor, analysis of the *tpt* and *pht2; 1* mutant showed that both transporters contribute to stromal Pi levels in leaves. The TPT transporter contributes more to stromal Pi concentration in the adaxial mesophyll, while PHT2; 1 contributes more in the abaxial mesophyll. Low stromal Pi levels reduce photosynthetic efficiency (ΦPSII) and increase photoprotective NPQ. ADE, adaxial epidermis; ADM, adaxial mesophyll; ABM, abaxial mesophyll; ABE, abaxial epidermis; CFP, cyan fluorescent protein. Confocal images were kindly given by Prof. Wayne K. Versaw. The leaf cross-section image was modified from Subramani Raju et al. (2024). Created by BioRender.com.

In this issue of *Plant Physiology*, Subramani Raju et al. 2024 used a FRET (Förster resonance energy transfer)-based Pi sensor to evaluate the role of TPT, PHT2; 1, and PTH4; 4 as chloroplastic Pi transporters in *Arabidopsis thaliana* (Fig.). The authors estimated stromal Pi levels in Arabidopsis *tpt*, *pht2; 1* and *pht4; 4* mutants and the wild-type (WT) line grown under Pi-replete conditions. The *tpt* and *pht2; 1* mutant showed reduced stromal Pi levels compared with WT plants, while even lower Pi levels were observed in the *tpt pht2; 1* mutant line. Since stromal Pi levels did not differ between the *pht4; 4* mutant and the WT, the authors focused on the *tpt* and *pht2; 1* line for further studies.

To determine if stromal Pi concentration varies among leaf tissues and at different times of the day, Subramani Raju et al. (2024) studied its levels in adaxial and abaxial epidermis and mesophyll cells and at different time points spanning the photoperiod. The authors observed that stromal Pi levels vary during the day in the mesophyll but not in epidermal tissues. Moreover, measurements performed with the *tpt* and *pht2; 1* mutant demonstrated that these transporters contribute to stromal Pi level fluctuations and that this contribution was additive. Interestingly, the TPT transporter contributes more to stromal Pi levels in the adaxial mesophyll, while PHT2; 1 contributes more in the abaxial mesophyll (Fig.). Complementation of these mutants with their corresponding WT genes restores stromal Pi levels, establishing a causal link between the studied transporters and the low stromal Pi phenotypes. Using *TPT* and *PHT2; 1::mCherry* complemented lines, the authors confirmed that stromal Pi variation was not due to changes in the transporters’ protein abundance. Consequently, they suggest post-translational regulation of their activity during the photoperiod.

Phosphorus starvation impairs photosynthesis, an effect attributed to reduced stromal Pi levels (Carstensen et al. 2018). However, the inability to measure intracellular Pi concentration makes it difficult to understand its role and relevance in photosynthetic regulation. The experimental setup of Subramani Raju et al. (2024) allows for addressing this issue without the pleiotropic effects of imposing nutritional deprivation on the whole plant. Analysis of the *tpt* and *pht2; 1* mutant showed that reduced stromal Pi levels decrease the quantum yield of PSII (ΦPSII) and increase Non-Photochemical Quenching (NPQ) under steady-state growth conditions, confirming a correlation between stromal Pi and photosynthetic performance.

NPQ can be induced through activation of the xanthophyll cycle and by acidification of the thylakoid lumen (Müller et al. 2001). Low stromal Pi levels can trigger the latter due to reduced ATP synthase conductivity of protons. To test this hypothesis, the authors measured proton conductivity (g_H_^+^) and proton motive force (*pmf*) using electrochromic shift. No differences in g_H_^+^ or *pmf* were observed between mutants and WT plants under steady-state growth conditions. However, both parameters were transiently reduced in the *tpt* and *pht2; 1* mutant during dark-to-light transitions, returning to WT levels within minutes of light exposure. Hence, thylakoid *pmf* and ATP synthase activity acclimate to reduced stromal Pi levels during the onset of illumination. This acclimation response persists during steady-state growth conditions and affects ΦPSII and NPQ. The nature of this acclimation mechanism remains unknown and may involve an alternate mechanism of lumen acidification and changes in the ATP synthase Pi affinity or its active pool size.

In summary, Subramani Raju et al. (2024) advances our understanding of chloroplast Pi transport and the effect of stromal Pi on photosynthetic regulation. The authors characterized stromal Pi variations in different leaf tissues and throughout the photoperiod and the relative contribution of the TPT and PHT2; 1 transporter to stromal Pi concentration. Their FRET-based sensor allows studying the relation between subcellular Pi and photosynthetic performance without the pleiotropic effects associated with P deprivation treatments. Results suggest that novel regulatory mechanisms participate in photosynthetic acclimation to low stromal Pi concentration. Overall, the work performed by Subramani Raju et al. (2024) opens new possibilities for studying the effect of subcellular Pi concentration on plant performance.
